# Genetic and Molecular Analysis of Essential Genes in Centromeric Heterochromatin of the Left Arm of Chromosome 3 in *Drosophila melanogaster*

**DOI:** 10.1534/g3.119.0003

**Published:** 2019-04-04

**Authors:** Monika Syrzycka, Graham Hallson, Kathleen A. Fitzpatrick, Inho Kim, Shawn Cotsworth, Rob E. Hollebakken, Kevin Simonetto, Linda Yang, Stephanie Luongo, Kevin Beja, Alistair B. Coulthard, Arthur J. Hilliker, Donald A. Sinclair, Barry M. Honda

**Affiliations:** * Department of Molecular Biology and Biochemistry (MBB), Simon Fraser University, 8888 University Dr, Burnaby BC V5A 1S6 and; ^†^ Dept. of Biology, York University, Toronto ON, M3J 1P3, Canada

**Keywords:** centromeric heterochromatin, essential genes, functional annotation

## Abstract

A large portion of the *Drosophila melanogaster* genome is contained within heterochromatic regions of chromosomes, predominantly at centromeres and telomeres. The remaining euchromatic portions of the genome have been extensively characterized with respect to gene organization, function and regulation. However, it has been difficult to derive similar data for sequences within centromeric (centric) heterochromatin because these regions have not been as amenable to analysis by standard genetic and molecular tools. Here we present an updated genetic and molecular analysis of chromosome 3L centric heterochromatin (3L Het). We have generated and characterized a number of new, overlapping deficiencies (Dfs) which remove regions of 3L Het. These Dfs were critically important reagents in our subsequent genetic analysis for the isolation and characterization of lethal point mutations in the region. The assignment of these mutations to genetically-defined essential loci was followed by matching them to gene models derived from genome sequence data: this was done by using molecular mapping plus sequence analysis of mutant alleles, thereby aligning genetic and physical maps of the region. We also identified putative essential gene sequences in 3L Het by using RNA interference to target candidate gene sequences. We report that at least 25, or just under 2/3 of loci in 3L Het, are essential for viability and/or fertility. This work contributes to the functional annotation of centric heterochromatin in *Drosophila*, and the genetic and molecular tools generated should help to provide important insights into the organization and functions of gene sequences in 3L Het.

Heterochromatin is the darkly-staining, densely-compacted chromatin of higher eukaryotes that occupies defined positions in homologous chromosomes (Heitz 1928, 1929). Constitutive heterochromatin is generally found near centromeres and in telomeres, remains densely packaged throughout the cell cycle, and consists largely of moderately and highly repetitive sequences (Lohe *et al.* 1993; Hoskins *et al.* 2007; Smith *et al.* 2007).

In *Drosophila melanogaster*, about 60 Mb of DNA (roughly 1/4 of the genome) in females is found within heterochromatic regions, while in males, the 40Mb Y chromosome is almost entirely heterochromatic (Adams *et al.* 2000; Hoskins *et al.* 2002). However, while there has been extensive genetic and molecular characterization of genes in *Drosophila* euchromatin, genes in the heterochromatic regions of the genome have been much more difficult to study. Some obstacles to mapping and assembling heterochromatic sequences include an absence of significant meiotic recombination, a paucity of prominent cytological landmarks, and the high repetitive sequence content within heterochromatin.

A defining property of heterochromatin is its ability to variably “silence”, in a mosaic fashion, euchromatic genes relocated immediately next to or within heterochromatin, a phenomenon called position effect variegation (PEV) (reviewed in Eissenberg and Reuter 2009). Genetic screens for second-site suppressors and enhancers of PEV have identified a large number of modifier genes, many of which encode known enzymatic products or structural components associated with establishment/maintenance of heterochromatin (Eissenberg *et al.* 1990; Reuter *et al.* 1990; Tschiersch *et al.* 1994; Schotta *et al.* 2004). Moreover, heterochromatic regions often contain signature patterns of histone modifications and bound proteins similar to those found in some silenced euchromatic gene regions, including the presence of HP1a, Su(var)3-9 and Su(var)3-7 proteins, as well as histones trimethylated at residues H3K9 and H4K20 (Kharchenko *et al.* 2011; Riddle *et al.* 2011).

Although heterochromatin has striking gene silencing properties, genetic analysis has demonstrated that these chromosomal regions contain active genes whose expression is essential for fly development and fertility. A large number of these genes reside in the centromeric heterochromatin of the autosomes–chromosome 2 (Hilliker and Holm 1975; Hilliker 1976; Myster *et al.* 2004; Coulthard *et al.* 2010) and chromosome 3 (Marchant and Holm 1988a,b; Schulze *et al.* 2001, 2005). In addition, a few essential genes are located on the X (Hilliker and Appels 1982; Mével-Ninio *et al.* 2007) and Y (Carvalho *et al.* 2015; Chang and Larracuente 2018) chromosomes, as well as a number on 4, the “dot” chromosome, which has several properties consistent with a heterochromatic environment (Riddle and Elgin 2018).

It is interesting that heterochromatic genes can in turn be repressed when placed in euchromatic locations, strongly suggesting that these genes require a heterochromatic environment for their expression (Wakimoto and Hearn 1990; Eberl *et al.* 1993; Howe *et al.* 1995). This hypothesis is further supported by several genetic and molecular studies which suggest that reduced dosage of the HP1a gene, which encodes a product required for heterochromatin integrity, leads to the reduced expression of some heterochromatic genes (Clegg *et al.* 1998; Sinclair *et al.* 2000; Lu *et al.* 2000; Schulze *et al.* 2005).

The first genetic screens for lethal mutations in chromosome 3 pericentric heterochromatin identified a minimum of 10 essential loci in 3L Het and 2 loci in 3R Het (Marchant and Holm 1988a and b). Subsequent genetic analysis generated more 3L Het deficiencies, additional alleles of already defined 3L Het genes, as well as mutations in new essential 3L and 3R Het loci, some of which were identified molecularly (for a review, see Fitzpatrick *et al.* 2005).

Data providing a corresponding physical map of chromosome 3 centric heterochromatin has come from genome sequencing as well as from cytological studies., Progress from the Drosophila Heterochromatin Genome project has provided an extensive physical map from DNA sequencing data, including numerous predicted gene models in centric heterochromatin (Smith *et al.* 2007; Hoskins *et al.* 2007, 2015). Comprehensive ChIP-array analysis of histone modifications and chromosomal proteins has also demonstrated different chromatin packaging profiles for heterochromatic *vs.* euchromatic domains (Riddle *et al.* 2011).

Koryakov *et al.* (2002) used various chromosomal rearrangements and heterochromatin banding analysis to produce a cytological map of the essential 3L and 3R Het loci defined by Marchant and Holm (1988b). This group later refined their map using the *Suppressor of Under-Replication* (*SuUR*) mutation, which allows visualization of chromatin landmarks in the otherwise rather amorphous heterochromatin (Koryakov *et al.* 2003). More recently, Andreyeva *et al.* (2007) used FISH mapping of BAC clones containing known essential 3L and 3R Het genes on polytene chromosomes from *SuUR- Su(var)3-9* double mutants to generate a high-resolution map of heterochromatic gene models and highly repetitive satellite sequences.

A comprehensive analysis of essential genes in the centric heterochromatin of chromosome 2 has been provided by extensive genetic and molecular analyses by Hilliker (Coulthard *et al.* 2003, 2010) and others (Myster *et al.* 2004). In particular, Coulthard *et al.* (2010) further refined the genetic and molecular profile of the distal segment of proximal 2R Het, reducing the number of complementation groups defined by Myster *et al.* (2004) and identifying six additional essential loci in 2R Het via RNAi analysis . Their results complement well the extensive physical map generated by genome sequencing (Hoskins *et al.* 2007, 2015) and cytological analysis (Rossi *et al.* 2007; Dimitri *et al.* 2009).

Here we report on our work to obtain a comprehensive genetic map of chromosome 3L Het. We focused on the identification of essential genetic loci and their alignment with predicted gene models on the physical map, with the overall aim of obtaining a functional annotation of essential genes in this region. This and our previous work has also facilitated the characterization of a number of interesting essential genes in centric heterochromatin *e.g.*, Schulze *et al.* (2005), Li *et al.* (2009), Hallson *et al.* (2008, 2012), Sinclair *et al.* (2009), as well as genes described in this report.

Briefly, we focused initially on compiling a collection of new genetic lesions, followed by genetic deficiency mapping and a large scale *inter se* complementation analysis to define genetic loci. Next, we used a PCR-based mapping approach to localize putative heterochromatic sequences from the available genome assembly to defined heterochromatic intervals, thereby allowing us to identify candidate gene(s) for each essential complementation group. We then identified essential loci present by correlating gene sequences to lethal complementation groups, coupled with RNA interference studies (see also Hallson *et al.* 2012 for examples). Most of the original lethal 3L Het complementation groups identified by Marchant and Holm have now been defined at the molecular level and we have also identified additional essential loci.

Our analysis has revealed a surprisingly high percentage of essential genes in 3L Het. The work also provides an expanded repertoire of genetic reagents, such as deletions (especially those with molecularly defined breakpoints), as well as novel lethal mutations (EMS-, *P*-element- and radiation-induced); these will be valuable tools for developing a comprehensive profile of gene functions in chromosome 3 heterochromatin.

## Materials and Methods

### Fly strains and crosses

Flies were raised on standard yeast-molasses-cornmeal medium and fly strains were maintained at 18°. Crosses were performed at 25° unless otherwise stated and all crosses were scored to completion. If no transheterozygous progeny emerged, or if the number of transheterozygous progeny was less than 5% of those expected from balancer classes (minimally, 100 balancer heterozygotes were examined per cross), the combination was considered lethal. In cases where the transheterozygotes were greater than 5% but substantially less than 50% of those expected, the combination was considered semi-lethal.

Unless otherwise stated, genetic strains used have been reported elsewhere. The G series of EMS-induced lines, as well as *Df(3L)FX3*, *DF(3L)FX53*, *Df(3L)FX33* and *Df(3L)MX18* were created in the Deitcher laboratory (Vilinsky *et al.* 2002; Schulze *et al.* 2005; D. Deitcher personal communication). The Z series of EMS mutations was obtained from the Zuker collection (Koundakjian *et al.* 2004). EMS lines *2-30* and *1-166-38*, as well as the γ-radiation-induced deficiencies *Df(3LR)6B-29*, *Df(3L)1-166*, *Df(3L)9-56*, *Df(3L)2-30* and *Df(3L)8-80* were generated by Marchant and Holm (1988 a). The *P*-element alleles *CH(3)4*, *CH(3)119*, *CH(3)4d*, *CH(3)53* and *CH(3)7* were generated by Zhang and Spradling (1994) and were generously provided by P. Zhang. *Df(3L)O-1*, *Df(3L)K2* and *Df(3L)γ-28* were generated as described in Schulze *et al.* (2001).

### X-ray mutagenesis and genetic screens

Three- to five-day old *KG03264/KG03264* males (carrying a P{SUPor-P} insert at cytological position 80C1 near the *nrm* locus) were treated with 4,000 rads of X-ray radiation, allowed to recover for 24 hr, and crossed en* masse* to TM3 Sb Ser**/TM6 Hu Tb** virgin females. Approximately one thousand single F1 KG03264{w^+^}*/TM3 Sb Ser** or KG03264{w^+^}*/TM6 Hu Tb** males (where * indicates a mutagenized third chromosome) were then crossed to virgin females carrying a 3L Het deficiency (*Df(3L)FX3*/TM3 Ser** or *Df(3L)MX18* /*TM3 Ser)*, and the resulting progeny were examined for lethality or visible phenotypes. Stocks of putative lethal mutations were established from KG03264{w^+^}*/TM3 Ser** siblings. To further map the newly-isolated lesions, all mutations were separately crossed to flies bearing a subset of smaller 3L Het deficiencies (*Df(3L)1-166*, *Df(3L)γ28*, *Df(3L)K2*, *Df(3L)9-56*, *Df(3L)FX33*), and to at least two existing alleles of each lethal complementation group (except where only a single allele existed).

We then conducted a second X-ray screen, designed to isolate lethal lesions in both 3L Het and 3R Het. In this study, 3- to 5-day old *e^1^/e^1^* males were treated with 4,000 rads of X-ray radiation and crossed *en masse* to TM3 e^1^ Sb Ser**/TM6 e^1^ Hu Tb** virgin females. Single F1 e^1^*/TM3 e^1^ Sb Ser** males (where * indicates a mutagenized third chromosome) were then crossed to *Df(3LR)6B-29*/TM3 e^1^ Ser** females, and stocks of putative lethal mutations established from e^1^*/TM3 e^1^ Ser** siblings. To further map the newly isolated lesions, we crossed all mutations to the subset of smaller heterochromatic deficiencies, as described above, with the addition of *Df(3R)10-65* and *Df(3R)XM3*, as well as to alleles of each lethal complementation group.

### Screen of the EMS Zuker collection

We obtained 3,400 strains (balanced with TM6 e^1^ Tb**) from the Zuker collection that contained putative third chromosome mutations generated with EMS, because it had been reported that approximately one third of the Zuker strains contained third chromosome lethal mutations (Koundakjian *et al.* 2004). We tested each balanced line for lethality with different 3L or 3R Het Df/TM6 Tb** strains: *Df(3LR)6B-29*, *Df(3L)FX53*, *Df(3L)Delta1AK and Df(3R)XM3*. Confirmed lethal mutations were then further mapped by using *Df(3L)γ28*, *Df(3L)K2*, *Df(3L)9-56*, *Df(3L)FX33*, *Df(3R)XM3* and *Df(3R)10-65*, each balanced over TM6B Tb**. In addition, we tested the lethals for allelism with representative alleles of appropriate lethal complementation groups.

### Single embryo PCR for detection of homozygous mutant embryos, deficiency mapping, and sequencing of mutant alleles

We isolated embryos homozygous for chromosomal deficiencies and other induced mutations using the methodology described in Hallson *et al.* (2008; 2012). In brief, using DNA from homozygous deficiency strains, along with PCR mapping primers for candidate gene sequences (see Table S1), we tested for the presence or absence of PCR products in homozygous mutant embryos from each strain. The absence of a PCR product indicated that a specific gene sequence mapped to the region covered by the deficiency.

For sequence analysis, DNA was isolated from homozygous mutant embryos, all exon regions were PCR amplified, and purified PCR products were then sequenced to look for corresponding DNA lesions that might result in mutant phenotypes (see Hallson *et al.* 2008, 2012). DNA sequencing was performed by UBC NAPS or Genewiz or Macrogen sequencing services. Mutations were identified by comparing sequences to the reference sequences available on Flybase using the BLAST algorithm (Altschul *et al.* 1990) and multiple protein alignments were carried out using CLUSTALW (Larkin *et al.* 2007).

### RNA interference experiments

Transgenic UAS-RNAi lines, expressing inverted repeats (IRs) under the control of *Gal4*, were obtained from the Vienna Drosophila RNAi Center (VDRC: http://stockcenter.vdrc.at/control/main), Drosophila Transgenic RNAi Project (TriP: http://www.flyrnai.org/TRiP-HOME.html) and the Fly Stocks of the National Institute of Genetics (NIG-fly; http://www.shigen.nig.ac.jp/fly/nigfly/index.jsp). We crossed UAS-IR-bearing males to virgin females, initially held at 29° for at least two days, bearing *UAS-Dcr-2/UAS-Dcr-2*; tub-Gal4/TM3 Sb**. Actin and tubulin driver stocks were used to induce ubiquitous expression of the IR transgenes and a copy of *UAS-Dcr2* (a component of the RNAi pathway) was included to sensitize the RNAi interference pathway and enhance knockdown. We maintained crosses at 29° and then examined them for lethality, sterility and visible phenotypes in flies expressing the IR sequence. Crosses producing UAS-IR driven progeny, but at proportions substantially less than 50% of those expected from the balancer classes, were deemed semi-lethal (due to the incomplete penetrance/ expression of RNA, some “escapers” may survive).

### Data Availability

A comprehensive and representative set of stocks have been sent to Dr Kevin Cook at the Bloomington Drosophila stock center. Supplemental files available at FigShare. Table S1 lists primers used in mapping. Table S2 lists EMS mutations identified. The authors affirm that all data necessary for confirming the conclusions of the article are present within the article, figures, and tables. Supplemental material available at Figshare: https://doi.org/10.25387/g3.7828301.

## Results

As a first step in our analysis, we generated and mapped a large number of additional lethal lesions (putative Dfs and/or point mutations) within 3L and 3R Het. To do this, we conducted two X-ray mutagenesis screens, and in parallel screened 3,400 EMS-mutagenized 3^rd^ chromosomes from the Zuker collection (Koundakjian *et al.* 2004), as outlined in Materials and Methods.

From these large-scale genetic screens, we isolated 91 novel lesions (46 from the X-ray screens, 45 from the Zuker lines), and of these, 60 mapped to 3L Het and 14 to 3R Het (Table 1). The remaining 17 mutations may be located outside of 3^rd^ chromosome heterochromatin, possibly due to the presence of second site lethal mutations outside of 3L Het, see below.

**Table 1:** Summary of results from EMS and X-ray screens

Mutagen	3L lesions	3R Lesions	Total number of lesions	Number of chromosomes	Df used for screening	Frequency of lesions
X-ray #1	14	0	14	∼1000	FX3, MX18	1.40%
X-ray #2	25	7	32	3829	6B-29	0.84%
EMS	21	7	45*	3400	6B-29, Delta1-AK, FX53, MX3	1.32%

### Screens for lethal lesions in 3L and 3R Het induced by X-rays

**Figure 1:**
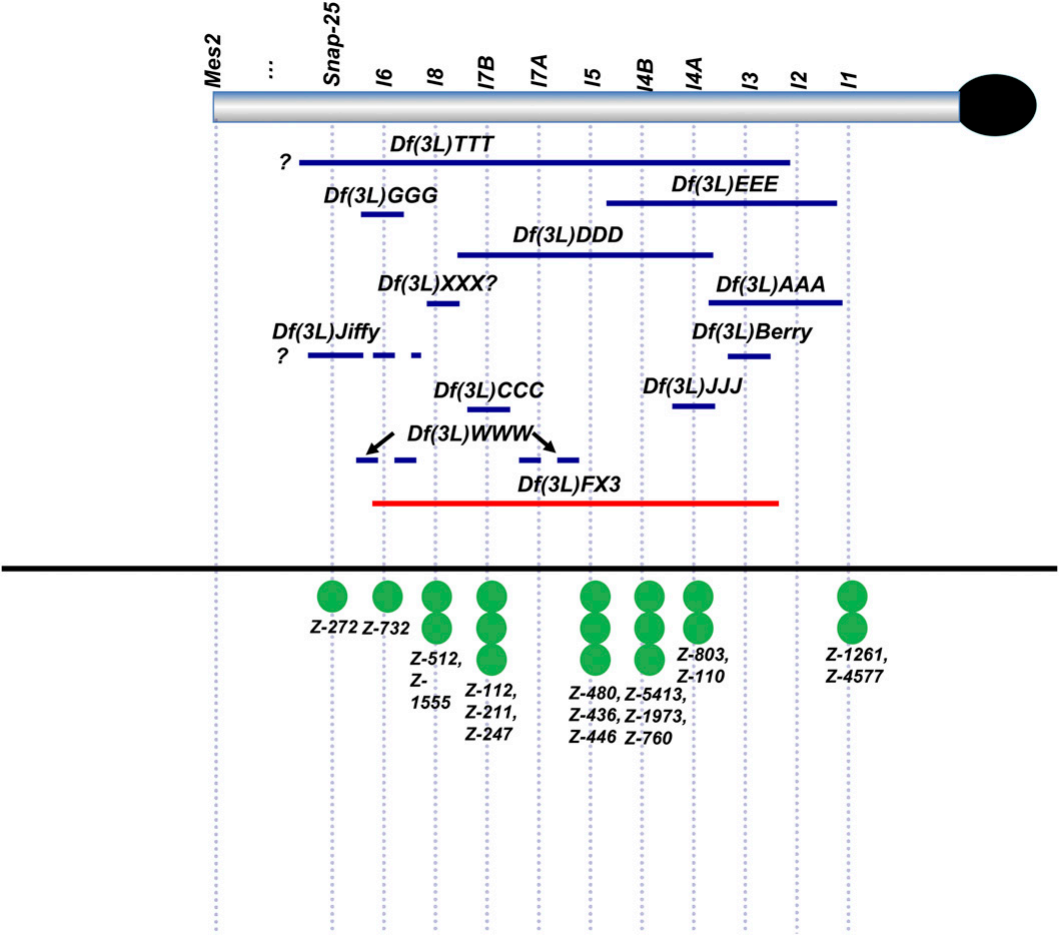
Genetic map of EMS mutants and deficiencies isolated from our initial screens of Zuker lines and first X-ray screen. Blue lines - newly discovered deficiencies; red lines - deficiencies used to screen for new lesions; green circles - newly-mapped EMS lesions from the Zuker collection. Dashed lines on deficiencies indicate semi-lethality with alleles of intersecting complementation groups. *Df(3L)WWW* may be a discontinuous lesion affecting at least two genes (*lethal 6* and *lethal 7A*).

Our first X-ray screen with the large *Df(3L)FX3* and *Df(3L)MX18* deficiencies identified 14 lesions in 3L Het (X-ray #1 in Table 1). To position the new lesions more definitively, we tested them for complementation with deficiencies removing specific intervals of 3L Het and with alleles of previously identified essential loci. Of the aforementioned 14 lesions, 11 failed to complement *Df(3L)FX3* (see Figure 1). The remaining three mutations (*Df(3L)Lola*, *Df(3L)BAC and Df(3L)ZZZ*) were *Minute*-like in phenotype in combination with *Df(3L)FX3* or *Df(3L)MX18*, but it was not possible to map these lesions further. The combined frequency of lethal and *Minute*-like lesions generated in this screen was 1.4%. The putative X-ray-induced lesions varied in size; some were large and spanned several lethal complementation groups, corresponding to many megabases of DNA (*e.g.*, *Df(3L)TTT*) (Figure 1), whereas others were either small deletions/other rearrangements or possibly point mutations affecting single genes (*e.g.*, *Df(3L)GGG*) (Figure 1).

**Figure 2:**
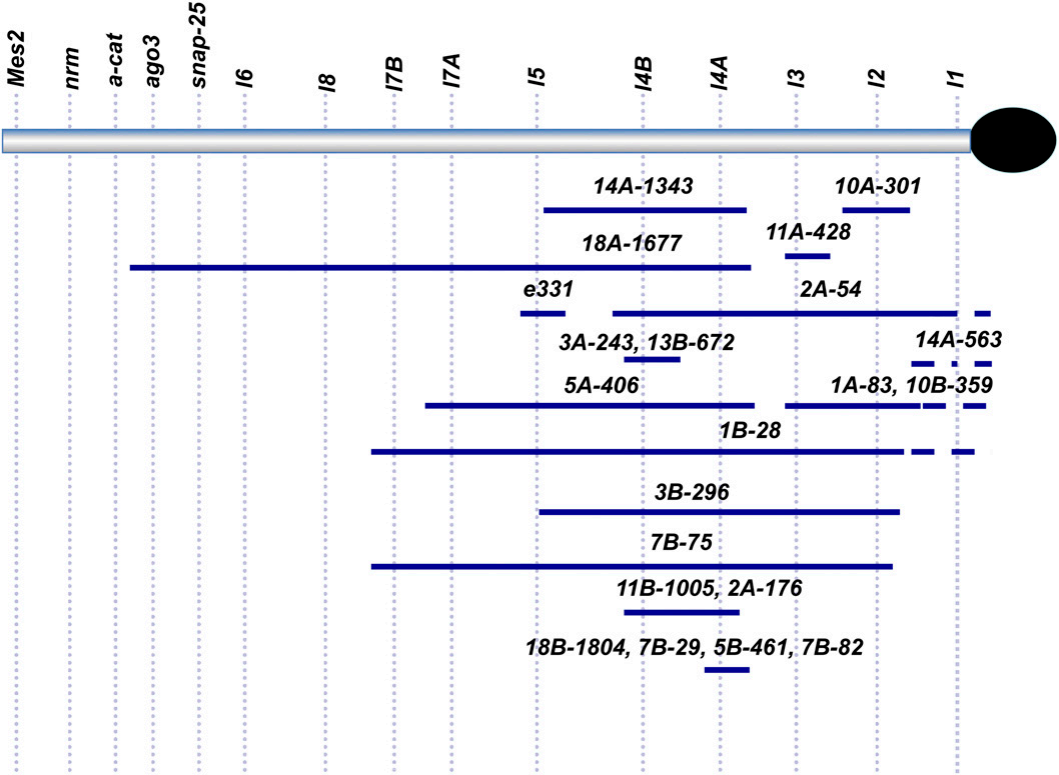
Map of deficiencies generated in a more intensive, second X-ray screen. Positioning was also obtained by crossing new deficiency strains to alleles affecting essential loci and other deficiencies. Note that of the 25 deficiencies isolated, 2 were not used further (not shown) and 1 was viable with alleles of all representative complementation groups we tested in 3L Het.

In a subsequent larger-scale X-ray screen (X-ray #2 in Table 1), we screened the mutagenized chromosomes for failure to complement *Df(3LR)6B-29*, a large deficiency removing heterochromatic segments from both 3L and 3R (Marchant and Holm *op. cit*.). In total, we isolated 32 lesions in this second screen: 25 on 3L and 7 on 3R. Of the 25 putative 3L Het deficiencies isolated in our second X-ray screen, 21 were used for further analysis (see Figure 2). We then performed *inter se* complementation tests of each of these lesions in combination with various other 3L Het deficiencies and in combination with representative alleles of existing complementation groups, in order to position the new lesions along the chromosome arm (see Figure 2 for a summary).

### Screening for lesions induced by EMS in 3L and 3R Het

As described in Materials and Methods, an initial screen of recessive lethal lines from the Zuker EMS collection (Koundakjian *et al.* 2004) involved testing each strain for lethality in combination with *Df(3LR)6B-29*, a deficiency that removes much of chromosome 3 heterochromatin (Marchant and Holm 1988a). All putative 3L and 3R Het lethal mutations identified in this analysis were tested with four other deficiencies that remove segments of 3^rd^ chromosome centric heterochromatin (*Df(3L)Delta1-AK*, *Df(3L)FX53*, *Df(3L)FX3*, and *Df(3R)XM3)*. Of the 45 3L Het EMS lesions identified (Table 1), 44 were lethal or semi-lethal in combination with one of the four deficiencies. The single lesion (*Z-4493*) that was viable *in trans* with all four deficiencies was characterized by a curly wings phenotype when tested in combination with *Df(3R)XM3*. Three other lesions also caused visible phenotypes (black deposits at leg joints, abnormal positioning of legs and curled wings) and were also semi-lethal when hemizygous over particular deficiencies.

Of the 45 EMS mutations identified (designated with an asterisk in Table 1), 21 could be assigned unequivocally to 3L Het, based on complementation tests with *Df(3L)FX53*, *Df(3L)Delta 1-AK* or *lethal 1* alleles (see Table S2). Seventeen of these 21 lesions were allelic with previously identified complementation groups, 2 failed to complement *Df(3L)Delta 1-AK* (distal to *SNAP-25*), 1 (*Z-855*) was semi-lethal in combination with *Df(3L)FX53*, and 1 failed to complement *Df(3L)K2* and *Df(3L)1-166*. An additional seven lesions were found to be allelic with complementation groups in 3R Het.

None of the remaining 17 lesions could be assigned to known lethal complementation groups in either 3L or 3R Het. It is likely that at least some of the additional mutations are allelic to second-site mutations carried by the deletion chromosomes used for complementation analysis, as accumulation of lethal mutations on permanently heterozygous chromosomes has been well-documented (Mukai 1964).

Our analyses revealed a few inconsistencies with respect to the previously published 3L Het map (Fitzpatrick *et al.* 2005), as well as one erroneous allele assignment. Our revised genetic map now places *Snap-25* and *lethal 6* distal to *lethal 8*, which in turn was placed distal to *lethal 7A* and *7B* (Figure 2). This gene order is now consistent with the physical map provided by genome sequencing (Hoskins *et al.* 2015), and this order was also confirmed by our mapping and sequencing of lethal complementation groups corresponding to these published gene models. Finally, allele *G1e* was previously incorrectly assigned to *lethal 7B* rather than *lethal 4B*.

### Refining the 3L Het physical map

In order to align the genetic and physical maps of 3L Het, we mapped candidate essential genes to specific genetic intervals as defined by our deficiency maps. Using DNA from homozygous deficiency strains and primers for a set of candidate genes, we tested for the presence or absence of PCR products in flies homozygous for a given deficiency strain. The absence of a PCR product indicated that a specific gene sequence was removed by the deficiency. We then refined our analysis by using reference sequences in the physical map as anchor points for our genetic map, limiting the number of candidate genes required for testing with each deficiency.

**Figure 3:**
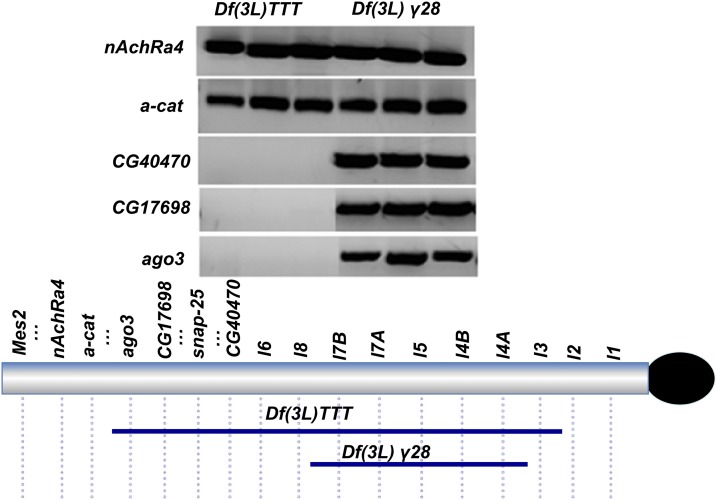
Determining the distal extent of *Df(3L)TTT* by gene-specific PCR. *CG40470* is a gene located between *SNAP-25* and *l6*. Prior genetic analysis confirmed that *Df(3L)TTT* failed to complement alleles of essential genes located in the 3L Het segment extending from *lethal 3* to *SNAP-25*. *Df(3L)gamma-28*, which does not delete the regions tested, was used as a positive control.

**Figure 4:**
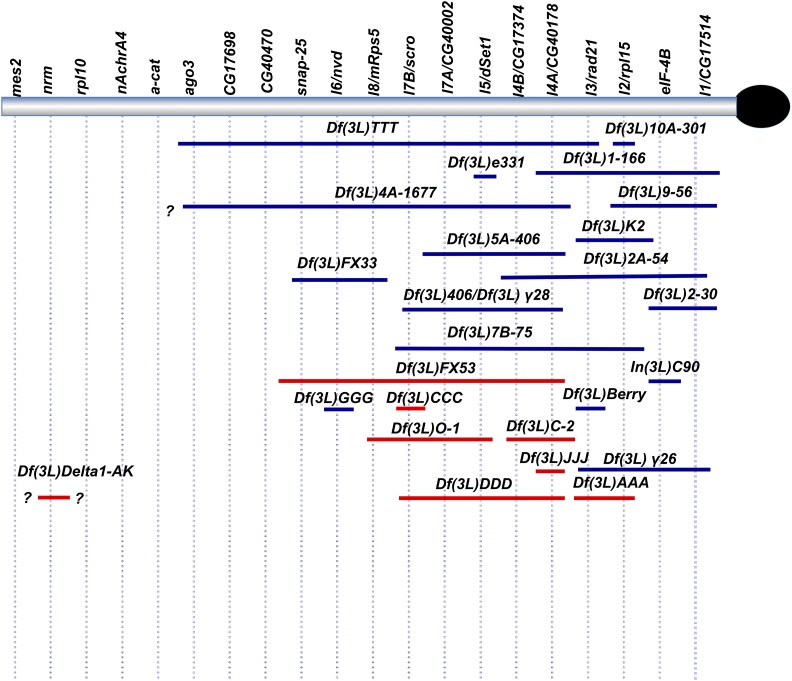
Refined map of 3L Het incorporating genetic and molecular data from this study. Blue marks deficiencies in which extent was determined genetically and molecularly. Red marks deficiencies in which limits were determined genetically. “?” denotes boundary limits that have not been molecularly defined.

In order to better align the genetic complementation map to the published heterochromatic sequence in Release 6 (Hoskins *et al.* 2015), we also mapped endpoints of several large and/or distal deficiencies by PCR relative to 3L Het candidate gene sequences. Since to date, few mutations have been mapped to the distal segment of 3L Het (*e.g.*, between *Snap-25* and *Mes 2*), we were especially interested in determining the distal-most extent of *Df(3L)TTT*. PCR analysis of homozygous *Df(3L)TTT* embryos showed that this deficiency removes the *CG40470*, *CG17698* and *ago3* loci, but not the *α-catenin* or the *n-AchRA4* genes (Figure 3). From our complementation analysis and single embryo PCR mapping data, we conclude that *Df(3L)TTT* deletes a region predicted to contain at least 3 Mb of DNA. A summary of our refined map is presented in Figure 4.

### Correlation of gene models with lethal complementation groups

Our molecular analysis of deficiency strains combined with deletion mapping of essential genes allowed us to narrow the number of candidate genes that could potentially correspond to each genetically identified locus on our map. In an attempt to determine the molecular identity of each vital locus, we amplified and sequenced the entire coding region of each candidate gene from DNA homozygous for mutant alleles representative of a given complementation group. The presence of a significant sequence change (*e.g.*, predicted deleterious effects on gene product) relative to the control background sequence, led us to conclude that a given complementation group corresponded to the candidate gene in which the change was found. In certain cases, we were also able to rescue flies containing appropriate mutations transheterozygous to deficiencies removing the candidate gene by expressing a *Gal4* driven cDNA transgene of the corresponding gene of interest. Essential genes identified using this method are listed in Table 2; with putative or confirmed functions of these genes included in Table 6. Taken together with the RNAi results described below, the data from these analyses have allowed us to assign a molecular identity for each lethal complementation group present on our genetic map (shown in Figure 4).

**Table 2:** Known lethal complementation groups in 3L Het, and their molecular assignment based on the DNA lesions identified in mutants. (*These designations based on those used in Marchant and Holm 1988a,b). More comprehensive data for lethal 6 (*nvd*) will be reported in Syrzycka *et al*. manuscript in preparation

*Lethal complementation group:	Mutant Allele(s):	Mutagen:	Gene Affected:	Resulting change:	Source
lethal 1	In(3L)C90	γ-ray	CG17514	Genes missing	Marchant and Holm (1988b)
			eIF-4B/CG10837		
	Df(3L)2-30	γ-ray	eIF-4B/CG10837	Gene missing	Marchant and Holm (1988b)
	Z-4577	EMS	CG17514	W1092 > STOP	Zuker collection
					(Koundakjian *et al.* 2004)
	Z-1261	EMS	unidentified	unidentified	Zuker collection
					(Koundakjian *et al.* 2004)
lethal 2	See Schulze *et al.* (2005)	Various	RpL15/CG17420	See Schulze *et al.* (2005)	Schulze *et al.* (2005)
lethal 3	See Hallson *et al.* (2008)	Various	*rad21/vtd* (cohesin) CG17436	See Hallson *et al.* (2008)	Hallson *et al.* (2008)
lethal 4A	CH(3)53	P-element	CG40178	P insertion in intron	Zhang and Spradling (1994)
	CH(3)4d	P-element	CG40178	P insertion in intron	Zhang and Spradling (1994)
	Z-110	EMS	CG40178	L326 > STOP	Zuker Collection
					(Koundakjian *et al.* 2004)
	G8	EMS	CG40178	W377 > STOP	Deitcher laboratory
					(Vilinsky *et al.* 2002)
	G47	EMS	CG40178	K171 > STOP	Deitcher laboratory
					(Vilinsky *et al.* 2002)
	G6	EMS	CG40178	K180 > STOP	Deitcher laboratory
					(Vilinsky *et al.* 2002)
lethal 4B	G10	EMS	CG17374	A1463 > T	Deitcher laboratory
					(Vilinsky *et al.* 2002)
	G11	EMS	CG17374	R1542 > STOP	Deitcher laboratory
					(Vilinsky *et al.* 2002)
	G30	EMS	CG17374	G1780 > E	Deitcher laboratory
					(Vilinsky *et al.* 2002)
	Z-5413	EMS	CG17374f	G1779 > R	Zuker Collection
					(Koundakjian *et al.* 2004)
	Z-760	EMS	CG17374	I240 > S	Zuker Collection
					(Koundakjian *et al.* 2004)
	Z-1973	EMS	CG17374	A1060 > V	Zuker Collection
					(Koundakjian *et al.* 2004)
	3#69	EMS	CG17374	R67 > S	D. Sinclair
					(unpublished)
lethal 5	See Hallson *et al.* (2012)	Various	*Set1*/CG40351	See Hallson *et al.* (2012)	Hallson *et al.* (2012)
lethal 7A	fsa^2^l(3)	EMS	CG40002	Gene missing	Kennison and Tamkun
					(unpublished)+
lethal 7B	G43	EMS	Scro/CG17594	L250 > STOP	Deitcher laboratory
					(Vilinsky *et al.* 2002)
	Z-211	EMS	Scro/CG17594	P insertion of “A” nucleotide leading to frameshift at a.a. 293	Zuker Collection
					(Koundakjian *et al.* 2004)
	Z-247	EMS	Scro/CG17594	P insertion of “A” nucleotide leading to frameshift at a.a. 293	Zuker Collection
					(Koundakjian *et al.* 2004)
lethal 8	K125	P-element	mRpS5/CG40049	P insertion in intron 2	Current study
	Z-1555	EMS	mRpS5/CG40049	ATG to ATA	Zuker Collection
					(Koundakjian *et al.* 2004)
	Z-512	EMS	mRpS5/CG40049	G201 > E	Zuker Collection
					(Koundakjian *et al.* 2004)
	G18	EMS	mRpS5/CG40049	No lesion detected	Deitcher Laboratory
					(Vilinsky *et al.* 2002)

While most of the gene assignments summarized in Tables 2 and 6 were straightforward, the molecular and genetic characterizations of *lethal 1* and *lethal 7A* were slightly more complicated; these are therefore described in more detail below.

### Molecular characterization of lethal 1

**Figure 5:**
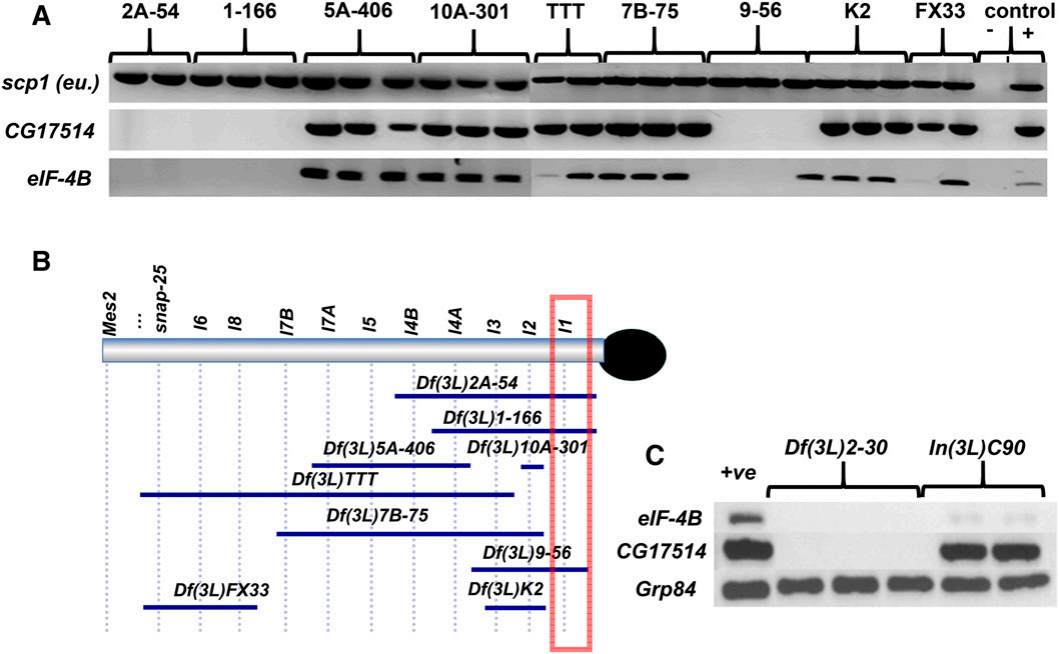
Mapping of elF-4B and CG17514**. A) Molecular positioning *eIF-4B* and *CG17514* in the lethal 1 region. B) *CG17514* and *eIF-4B* are deleted by *Df(3L)2-30*, and *eIF-4B*, but not *CG17514*, is deleted in the *In(3L)C90* strain.

*lethal 1* is the proximal-most genetically identified essential locus in 3L Het (see Figures 4 and 5). The locus was originally identified in mutagenesis screens conducted by Marchant and Holm (1988b) and it has been defined as a putative *trithorax group (trxG*) gene (Schulze *et al.* 2001). Numerous *lethal 1* alleles were isolated by Marchant and Holm (1988) and Schulze *et al.* (2001), suggesting that it may encode a large mutagenesis target; however, only two of the original mutations remain: *Df(3L)2-30* and *In(3L)C90*. *Df(3L)2-30* is associated with a duplication of proximal heterochromatic material between h48 and h50 (Koryakov *et al.* 2002), but it also likely carries a small deletion not detected by cytological analysis. *In(3L)C90* is an inversion with a heterochromatic breakpoint near or within *lethal 1*, and a euchromatic breakpoint in 62D2-7 (Lindsley and Hardy 1992). We have also identified two additional putative *lethal 1* alleles, *Z-1261* and *Z-4577*, both of which fail to complement deficiencies that lack the gene. In combination, these two alleles produce a small number of sterile transheterozygotes with a *Minute*-like phenotype, and extra wing vein material (Table 3). The conventional *Minute* phenotype is haplo-abnormal and thus dominant, and is characterized by developmental delay, shorter and thinner bristles, roughened/reduced eyes, misrotated genitalia, and, often, semi-sterility; this phenotype is most often caused by defects in global protein synthesis (see Marygold *et al.* 2007).

**Table 3:** *lethal 1 inter se* complementation matrix. ^M^All transheterozygous progeny were *Minute*-like. % expected = number of observed progeny / number of expected progeny based on balancer classes (*i.e.*, O/E)*100%. *In addition to deleting lethal 1, Df(3L)9-56, Df(3L)1-166, Df(3L)e*#54 and *Df(3LR)6B-29* remove the *lethal 2* (*rpL15*) locus and are *Minute* because of this (data not shown)

	*Z -1261*	*% expected*	*Z-4577 (W1092STOP)*	*% expected*
Df(3LR)6B-29*	20^M^ / 162 total	49%	2 ^M^ / 195 total	4%
Df(3L)1-166*	11^M^ / 148 total	30%	8 ^M^ / 182 total	18%
Df(3L)9-56*	1^M^ / 111 total	4%	0 / 186 total, *i.e.*, LETHAL	0%
Df(3L)e*#54*	5^M^ / 214 total	9%	5 ^M^ / 249 total	8%
***In(3L)C90***	1^M^ / 129 total	3%	19 ^M^ / 280 total	27%
***Df(3L)2-30***	24^M^ sterile / 203	47%	11^M^ sterile / 147 total	30%
***Z- 4577***	6^M^ sterile / 229 total	11%	Homozygous lethal	N*/*A
***Z- -1261***	Homozygous lethal	N*/*A	6^M^ sterile / 229 total	11%

In single embryo PCR mapping (Figure 5A) two potential candidate genes were absent from *Df(3L)2-30*: *eIF-4B (eukaryotic Initiation Factor 4B* or *CG10837)*, a member of the eukaryotic initiation factor complex required for translational activation, and *CG17514*, a large translation activator. However, *In(3L)C90* deletes *eIF-4B* but not *CG17514* (Figure 5C). This was somewhat surprising to us, because *lethal 1* is quite mutable, and because *CG17514* encodes a much longer protein (2,630 aa) than *eIF-4B* (459 aa); it therefore seemed more likely to us that *lethal 1* would correspond to *CG17514*. One possible explanation for the presence of *CG17514* exon sequences in *In(3L)C90* could be that only regulatory regions are mutated, and thus *CG17514* expression could still be affected.

Indeed, subsequent sequence analysis of mutant alleles provided evidence to identify *CG17514* as corresponding to *lethal 1*: the EMS-induced *lethal 1* allele *Z-4577* contains a mutation predicted to change of residue W1092 in the *CG17514* product to a stop codon, strongly suggesting that *CG17514* corresponds to the *lethal 1* locus.

In order to obtain additional support for our contention that *lethal 1* corresponds to *CG17514*, we carried out RNAi knockdown of *CG17514*; in this analysis, we crossed flies containing UAS responsive *CG17514* IRs to flies carrying the *tub-GAL4* transgene at 29° in order to activate targeting RNAi. Expression of 2 out of the 3 *CG17514* RNAi lines, VDRC 47269 and BL34355, resulted in reduced viability and complete lethality respectively. Escaper progeny expressing RNAi from the BDRC 47269 line display a *Minute*-like phenotype (thin bristles) and are sterile (Table 4). Taken together with the aforementioned data, these results indicate that *lethal 1* coincides with *CG17514*.

**Table 4:** RNAi experiments expressing *CG17514 RNAi*-activating *IRs* with *tub*-driven *GAL4* (at 29 C) suggest that *CG17514* is essential. However, viable progeny emerge when using the *eIF-4B RNAi* line (VDRC 31364). *Escapers emerging from the VDRC 47269 *CG17514* RNAi cross display a *Minute* phenotype and are sterile

Female parent	Balancer progeny	RNAi driven progeny	% of expected RNAi flies
***CG17514 RNAi / CG17514 RNAi (VDRC 47269)***	195	17*	9% (semi-lethal)
***CG17514 RNAi / CG17514 RNAi (BL34355)***	303	0	0% (lethal)
***CG17514 RNAi / CG17514 RNAi (VDRC 47268)***	151	105	63% (viable)
***eIF-4B RNAi /eIF-4B RNAi (VDRC 31364)***	196	109	71% (viable)
***eIF-4B RNAi /CyO (BL57305)***	324	123	>100% (viable)

However, these results do not exclude an essential role for the *eIF-4B* gene as well, given its presumed key role in protein. It is quite possible that our screens did not isolate any mutations in this smaller target gene, although results from RNAi experiments do not suggest that eIF-4B is essential either (see below).

### Molecular characterization of lethal 7A

**Figure 6:**
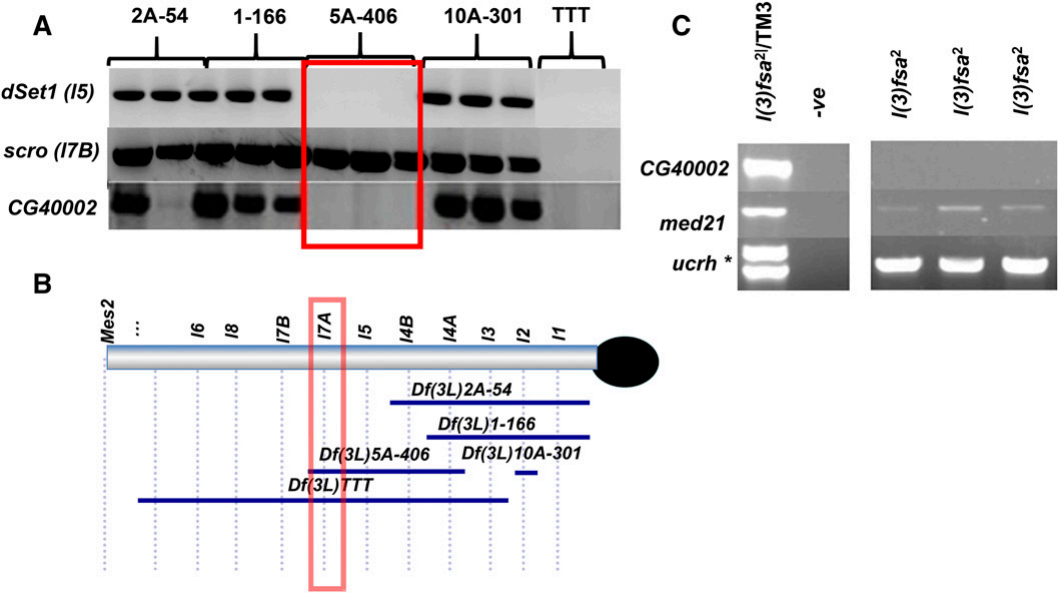
Mapping of *lethal 7A to CG40002*. A) Single embryo PCR mapping positions *CG40002* in the *lethal 7A* region. B) sePCR of DNA from *l(3)fsa^2^*mutants using *CG40002*, *med21* and *ucrh* primers. The asterisk marks a band that is amplified only from *l(3)fsa^2^*/*TM3* animals and likely corresponds to a polymorphic *ucrh* sequence found on this particular *TM3* balancer chromosome.

We currently possess only a single EMS induced allele of *lethal 7A*, *l(3)fsa^2^* (Fitzpatrick *et al.* 2005). This allele displays a lethal phase during late embryogenesis, with no obvious cuticle defects (Fitzpatrick *et al.* 2005). Molecular and genetic mapping show that *lethal 7A* is distal to *lethal 5* and proximal to *lethal 7B (scarecrow)* (see *e.g.*, Figure 6A). Two gene models, CG40002 and CG40472 (an apparent pseudogene of CG40002), appear the most likely candidates for *lethal 7A*. The *CG40002* gene encodes a 94 a.a. protein that is a member of the NADH dehydrogenase complex (a.k.a. Complex I). The NADH dehydrogenase complex is a conserved complex present throughout Eukarya and is the first major complex in the electron transport chain (Ohnishi *et al.* 2018). Given the important role of Complex I in the oxidative production of ATP, it would not be surprising if a gene encoding any of its subunits were essential.

There are also two other possible candidate gene models, *med21* and *ucrh*, in the vicinity. However, upon PCR mapping, we found that only the *CG40002* gene has been deleted in DNA from homozygous *l(3)fsa*^2^ mutant embryos, while *med21* and *ucrh* remain present (Figure 6C). This strongly suggests that *lethal 7A* corresponds to *CG40002*.

### RNAi knockdown of heterochromatic genes

Some 3L Het genes that were predicted to have important functions based on the existence of conserved human homologs did not yield mutant alleles in our screens. In order to test for additional essential genes in 3L Het, we performed RNAi knockdown analysis using relevant RNAi lines available from the VDRC, TRiP or NIG collections, which allowed us to target most 3L Het gene models. Each RNAi line was crossed to *UAS-Dcr2/UAS-Dcr2*; tub-Gal4/TM3 Sb** at 29°; *UAS-Dcr2* was used to optimize the RNAi pathway and therefore increase the likelihood of detecting gene knockdown effects. The results identify 9-10 additional essential genes in 3L Het (10 if *nAchRalpha4* is included, see Discussion below) not previously identified in mutagenesis studies (Tables 5 and 6). As positive controls, we used some RNAi transgenes targeting known lethal complementation groups, and these were found to be effective.

**Table 5:** Summary of RNAi experiments targeting genes in 3L Het. Column 1: The 3L Het genes targeted; Column 2: The identifier numbers of RNAi lines, appended with abbreviated prefixes indicating where they were sourced from (V - VDRC, BL - Bloomington Stock Center or NIG - National Institute of Genetics (Japan)); Column 3: Effects of driving RNAi on survival to adulthood and fertility (V - viable, L -lethal, SL - semi-lethal - *i.e.*, substantially less than 50% of expected progeny, F - fertile, S - sterile, IN - inconclusive, NT - not tested, M - male and FE – female % - for semi-lethal lines, percentage of RNAi progeny emerging relative to that expected from balancer classes); Column 4: Phenotypes observed in multiple *RNAi*-driven flies, where applicable; and Column 5: Predicted function based on homologous protein structure or functional studies

3L Het gene targeted (from proximal to distal)	RNAi lines used:	Effects of driving RNAi lines:	Phenotypes observed:	Encoded function targeted:
*CG17514/lethal1*	V47268, V47269, BL34355	V/F, SL (M: 9%, FE: 8%)/S, L	Thin thoracic bristles, tergite defects, body elongation, held out/blistered wings.	Translational activator.
*eIF-4B*	V31364, BL57305	V/F, V/F	Thin or missing thoracic bristles (V31364).	Translational initiation factor (may have an accessory role).
*dbp80*	BL3468, V109742	SL (M: 17%, FE: 18%)/S, L	Kinked bristles, tergite defects, wings held out or appear blistered.	RNA helicase. May be required for nuclear export of RNA.
*rpl15/lethal2*	not tested	N/A		Component of the ribosome.
*vtd/lethal3*	not tested	N/A		Sister chromosome cohesion, gene expression.
*CG40228*	V110000	SL (M: 11%, FE: 12%)/F	Ectopic thorax bristles, melanotic spots, flipped out wings.	Transcriptional elongation factor. Component of ELL?
*CG42598^%^*	V109076	V/F	Flies appear wider, segments elongated, flipped out wings.	Unknown.
*CG41284^%^*	V109076	V/F	Flies appear wider, segments elongated, flipped out wings.	Unknown.
*CG40160*	V109559, BL44268	L, V/F		Peptidase.
*CG40178/lethal4A*	V110089, BL44578	L, L		Thioredoxin, chaperone.
*FASN3 (CG17374)/lethal4B*	CG17374-2M, 3M	L, L		Fatty acid synthase.
*CG45782*	V109249, BL38367, V109094, BL40876	V/S (M only), V/F, L, L		Sucrose transporter.
*Ucrh/*UQCR-11	not tested	N/A		Oxidative phosphorylation.
*med21 (CG17397)*	BL34731, V31940 V109982, V13667	L, V, L, L		Transcriptional initiation, component of mediator.
*dSet1/lethal5*	V40682, V40683, V10833, V45267	L, L, L, L		H3K4 di- and tri-methylase.
*CG40002^%^*/*ND-AGGG*	V109239, BL43285	V/F, V/F		Oxidative phosphorylation.
*CG40472^%^*	nothing available	N/A		Oxidative phosphorylation.
*scro/lethal7B*	V33902	L		Cell-specific transcription factor.
*mRps5/lethal8*	BL36202	L		Mitochondrial ribosomal protein.
*nvd/lethal6*	not tested	N/A		Ecdysone biosynthesis, molting/metamorphosis.
*snap-25*	BL27306, BL34377	V/F, L		SNARE protein, exocytosis.
*CG40045*	V109167	V/F		E2 ubiquitin conjugating enzyme.
*CG40470*	not tested	N/A		Peptidase.
*CG43968*	nothing available	N/A		Unknown, may be secreted.
*CG17698*	V35634, V105884	V/F, L	Curved/kinked thoracic bristles.	Calmodulin dependent kinase, calcium signaling.
*CG40298*	nothing available	N/A		Unknown.
*CG17454*	V20458, BL43199	L, L		Spliceosome component.
*ago3*	not tested	N/A		piRNA biogenesis, germline silencing of transposons.
*alpha-Cat*	V19182, V20123	L, L		Cellular adhesion, adherens junction formation.
*CG32230/ND-MLRQ*	V9804, V10316, V101482	V/F, V/F, V/F	Melanin deposits on some flies.	Oxidative phosphorylation.
*nAChRa4*	V11392, BL31985, V12441	SL/F (M: 0%, FE: 9%), V/F, SL (M: 19%; FE: 29%)/F		n-acetylcholine receptor, neurotransmission.
*CG34031*	V109096	V/F		Unknown.
*vps11/CG32350*	V24731, V107420	L, V/F		Vacuolar protein sorting, lysosomes/late endosome function.
*CG33217*	V47818, V103309, BL43315, BL43167	L, L, L, L		Unknown.
*CKIIalpha*	not tested	N/A		Diurnal rhythm regulation, involved in cell signaling.
*rpL10/Qm*	V19083, V19084	L, L		Component of the ribosome.
*nrm*	V44176, BL52945	V/F, V/F		Adhesion at neuromuscular junctions.
*CG32457*	V15966, V15968	L, SL (M: 0%, FE: 10%)/F	Wings often not folded out in male escapers.	Unknown.
*CG32461*	V26149, V26150, V108971	V/F. V/F, V/F		Unknown.

^%^The sequences of these genes are very similar and are likely to result from annotation issues or more improbably, very recent duplications.

**Table 6:** Essential genes in 3L Het identified to date

Essential genes in 3L Het. (centromere outwards):	Determined essential based on:	Sources:
*CG17514/lethal1*	Lethality (muts/RNAi)/Male and fem. sterility (RNAi)	this study
*dbp80*	Male and fem. sterility (RNAi)	this study, Yan *et al.*, 2014
*rpl15/lethal2*	Lethality (muts)	this study, Schulze *et al.* 2005
*vtd/lethal3*	Lethality (muts/RNAi)	Hallson *et al.* 2008, this study (muts), Vass *et al.* 2003 (RNAi)
*CG40160*	Lethality (RNAi)	this study
*CG40178/lethal4A*	Lethality (muts/RNAi)	this study
*FASN3/lethal4B*	Lethality (muts/RNAi)	this study
*CG45782*	Male sterility (RNAi)	this study
*dSet1/lethal5*	Lethality (RNAi/muts)	Hallson *et al.* 2012, this study
*med21*	Lethality (RNAi)	this study
*CG40002/lethal7A*	Lethality (muts)	this study
*scro/lethal7B*	Lethality (RNAi/muts)	this study
*mRps5/lethal8*	Lethality (muts/RNAi)	this study, Yan *et al.*, 2014
*nvd/lethal6*	Lethality (muts/RNAi)	Yoshiyama *et al.* 2006 (RNAi), Syrzycka *et al.* in prep
*snap-25*	Lethality (muts/RNAi)	Vilinsky *et al.* 2002 (muts), this study (RNAi)
*CG17698*	Lethality (RNAi)	this study
*CG17454*	Lethality (RNAi)	this study
*ago3*	Female sterility (muts)	this study, Li *et al.* 2009 (muts)
*alpha-Cat*	Lethality (muts, RNAi)	Sarpal *et al.* 2012 (muts), this study (RNAi)
nAChRalpha4*	Lethality (RNAi)	this study
*CG32350*	Lethality (RNAi)	this study
*CG33217*	Lethality (RNAi)	this study
*CKIIalpha*	Lethality (muts)	Lin *et al.* 2002 (muts)
*rpL10/Qm*	Lethality (muts, RNAi)	Cook *et al.* 2012 (muts), this study (RNAi)
*nrm*	Lethality (muts)	Kania *et al.* 1993, Kania and Bellen 1995 (muts)
*CG32457*	Lethality/Sterility (RNAi)	this study

^%^The sequences of these genes are very similar and are likely to result from annotation issues or more improbably, very recent duplications.

* Potential essential gene identified based on failure to recover males in a single RNAi line. Note that this result is not entirely conclusive, as this RNAi line also has 1 predicted off-target.

### Summary of essential genes in 3L heterochromatin

**Figure 7:**
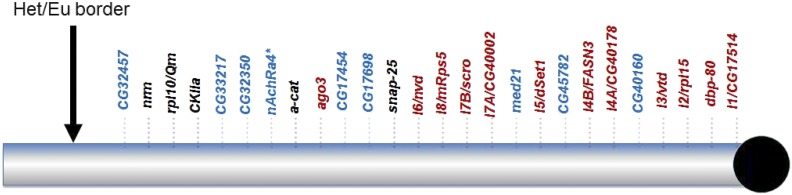
Map of essential genes present in 3L Het (not to scale). Blue- genes identified by us as essential through RNAi screening; red- genes identified by us as essential through mutants; black – genes identified by others as essential. *marks the uncertain status of *nAchRa4*, as discussed above.

The essential 3L Het genes identified either through isolation/analysis of mutant alleles or RNAi analysis are shown in Figure 7 and listed in Table 6. Overall, our data suggest that at least 25/39 genes (roughly 2/3) of genes in 3L Het are essential (26/39 if we include *nAchRalpha4* as a putative lethal), an unexpectedly large proportion relative to the roughly 1/3 of euchromatic genes that are predicted to be essential based on analyses of specific euchromatic regions (see Discussion). In addition, it is possible that our estimate of essential 3L genes is conservative and the number of essential genes in this region may be higher. We aren’t able to readily estimate whether more genes may have been missed in our mutagenesis screens (see Hilliker *et al.* 1981 for a discussion of this point), and there is currently an absence of genetic reagents (RNAi constructs, other) for studying some 3L Het gene models.

### Unassigned lethal mutations generated in the current study

A number of lethal mutations characterized in our study have not yet been assigned to known complementation groups but may be located in 3L Het. For example, the *Df(3L)XXX* allele from the initial X-ray screen fails to complement *Df(3L)γ-28*, but it does complement *Df(3L)1-166* and *Df(3L)FX33* (see Figure 4, *Df(3L)XXX* not shown on map), suggesting that an additional essential gene may reside between the lethal 7B /scarecrow** and *lethal 8* loci. There are also several EMS lesions that fail to complement *Df(3LR)6B-29*, but which have not been assigned to any known complementation group. While some of these may be alleles of second-site mutations on the *Df(3LR)6B-29* chromosome, outside of 3L Het, others may represent alleles of unidentified essential 3L Het loci *e.g.*, *Z-855* and *Z-5460* clearly map to 3L Het, but they complement alleles of all known essential 3L Het complementation groups. In addition, *Z-1326* and *Z-1605* were identified on the basis of failure to complement *Df(3L)Delta1-AK* and remain unassigned; however, while these alleles may correspond to distally located 3L Het genes, it is also possible that they are alleles of euchromatic genes at the heterochromatic/euchromatic border which have not yet been well characterized. Further work is necessary to determine the basis of all of the unassigned mutations; however, the next release of an updated *Drosophila* genome sequence should make it possible to identify and characterize more of these uncharacterized loci.

## Discussion

The work reported here links a detailed genetic map of 3L Het with the physical map available on Flybase. In the process, we have been able to assign molecular identities to most of our lethal complementation groups in 3L Het, which in the past have only been studied at a genetic level. Since full characterization of any genome is incomplete without an analysis of heterochromatin, our work provides an initial platform for the functional annotation of essential genes residing within these fascinating, yet poorly understood, regions of the genome. Our collection of mutant strains will be useful for studying the function of heterochromatic genes that have resisted characterization due to their location in a genetically less tractable region of the genome.

Using RNAi knockdown analyses, we have identified an additional group of essential 3L Het genes not identified in mutagenesis screens. Lethality associated with RNAi gene targeting provides good evidence that a gene is essential: the possibility of RNAi off-target effects seems unlikely because for all but one of the genes tested for RNAi knockdown effects, there were no detectable homologies to the 19 b.p. gene regions used in the RNAi transgene design (data not shown); moreover, off-targeting effects in flies have been reported as being very low (Dietzl *et al.* 2007). The single exception involved two VDRC RNAi lines targeting the *nAChRalpha4* gene; each of these exhibits a single predicted 19 b.p. off-target dsRNA; however, the majority of dsRNAs available for this gene do result in gene specific targeting, and therefore this single off-target RNA may be inconsequential. A homozygous viable allele of *nAChRalpha4* has recently been reported to exhibit circadian defects (Shi *et al.* 2014). However, since this allele is associated with a single amino acid substitution and was obtained in a screen for viable modifier mutations, it is likely not a null allele. If the effects on viability we observe by expressing *nAChRalpha4* RNAi lines result from direct gene-targeting, the *nAchRalpha4* protein is likely essential and may have additional biological roles.

There remain some 3L Het gene models for which we have neither RNAi lines nor mutants, so at this point it is not possible to define whether any of these genes are essential. Furthermore, the observed viability when expressing available RNAi lines for certain genes does not prove that these genes are non-essential; an RNAi line may be poorly-expressed or substantial maternal effects may allow survival and thus obscure the effects of RNAi targeting. Indeed, gene expression studies showed that several RNAi lines tested only modestly reduced expression of the target genes (data not shown). Finally, while it seems likely that eIF-4B should also be essential, based on its presumed cell function, it is noteworthy that our results, as well as other RNAi analyses to date (http://www.genomernai.org/v17/fullPagePhenotypes/gene/3355041) are not consistent with an essential role for this protein (although again, similar caveats apply).

In terms of other potential caveats, lethal phenotypes resulting from RNAi knockdowns may not indicate that a gene is essential, because in some cases, genetic mutations knocking out the same gene do not show a lethal phenotype. This relatively rare phenomenon has been attributed to other factors such as genetic compensation (Rossi *et al.* 2015, and refs therein).

Other strategies could be employed to determine whether any of the remaining 3L Het genes are essential. For example, since transposon insertions near genes of interest are becoming increasingly available, these could serve as starting points for generating imprecise excisions that affect adjacent genes. In addition, genetic screens for mutations lethal *in trans* to the distal-most 3L Het deficiencies (*Df(3L)TTT* or *Df(3L)1677)* may yield more lethal mutations between *ago3* and *RpL10*. Analysis of the latter 3L Het segment has proved difficult because deletions that remove the *RpL10* locus exhibit dominant semi-lethality and female sterility (K. Fitzpatrick unpublished observations). Lastly, with the innovations of CRISPR technology, plus the availability of newer targeted genetic reagents (*e.g.*, Mi{MIC} and Mi{Trojan-GAL4.0} alleles, see Flybase), it may be possible to extend the current work, although there could be issues with accessibility of DNA sequences when packaged in the chromatin environment of centric heterochromatin.

The findings from this and other studies indicate that although gene density is relatively low in centric heterochromatin, many genes located in this chromatin environment are functionally important. The essential genes identified in the current study appear to span a diverse set of molecular and developmental functions. We and others have noted that there is no correlation between developmental or other specific expression patterns and the location of genes within 3L and other centric Het regions. Although the size of an average heterochromatic gene is considerably larger than that of a typical euchromatic gene, neither is there any apparent correlation between heterochromatic gene size and whether a given gene is essential. While our mutagenesis experiments often produced mutations in larger 3L Het genes (based both on physical locus and gene product length), mutant alleles of some smaller essential genes (*rpl15* and *CG40002*) were also isolated, and several other small essential genes (such as *med21* and *CG17454*) were identified through RNAi experiments. Thus, although our results indicate an unexpectedly higher proportion of essential genes in 3L Het, there are no obvious properties that distinguish them from euchromatic genes.

As mentioned, our data suggest that approximately 2/3 of genes residing in 3L Het are required for developmental viability and/or adult fertility. In contrast, it is estimated that only about 1/3 of euchromatic genes are essential (Miklos and Rubin 1996; Ashburner *et al.* 1999; Dietzl *et al.* 2007; Chen *et al.* 2010). It remains to be determined whether the large proportion of essential genes in 3L Het is significant or results from the relatively small sample size: in 3R Het, only four of the 11 genes appear to be essential (our unpublished observations), while the overall proportion of essential genes in 2R and 2L Het remains to be established.

The work described here, and the genetic tools generated, provide a foundation for advancing our understanding of the organization, functions and regulation to genes in these unique chromosomal regions. Fly stocks from this work, including representative Dfs across 3L and 3R Het, lethal alleles of individual genes, as well as other reagents (cDNA transgene and RNAi lines) will be available from the Bloomington *Drosophila* Stock Center, Indiana.

## References

[bib1] Adams M. D. Celniker S. E. Holt R. A. Evans C. A. Gocayne J. D., 2000 The genomic sequence of *Drosophila melanogaster*. Science 287: 2185–2195. 10.1126/science.287.5461.2185 10731132

[bib2] Altschul S. F. Gish W. Miller W. Myers E. W. Lipman D. J., 1990 Basic local alignment search tool. J. Mol. Biol. 215: 403–410. 10.1016/S0022-2836(05)80360-2 2231712

[bib3] Andreyeva E. N. Kolesnikova T. D. Demakova O. V. Mendez-Lago M. Pokholkova G. V., 2007 High-resolution analysis of *Drosophila* heterochromatin organization using SuUR *Su(var)3–9* double mutants. Proc. Natl. Acad. Sci. USA 104: 12819–12824. 10.1073/pnas.0704690104 17640911 PMC1937550

[bib4] Ashburner M. Misra S. Roote J. Lewis S. E. Blazej R., 1999 An exploration of the sequence of a 2.9-mb region of the genome of *Drosophila melanogaster*: The Adh region. Genetics 153: 179–219. 10471707 10.1093/genetics/153.1.179 PMC1460734

[bib5] Carvalho A. B. Vicosoa B. Russo C. A. M. Swenor B. Clark A. G., 2015 Birth of a new gene on the Y chromosome of *Drosophila melanogaster*. Proc. Natl. Acad. Sci. USA 112: 12450–12455. 10.1073/pnas.1516543112 26385968 PMC4603513

[bib6] Chang C. H. Larracuente A. M., 2018 Heterochromatin-enriched assemblies reveal the sequence and organization of the *Drosophila melanogaster* Y chromosome. Genetics. 10.1534/genetics.118.301765 PMC6325706 30420487

[bib7] Chen S. Zhang Y. E. Long M., 2010 New genes in *Drosophila* quickly become essential. Science 330: 1682–1685. 10.1126/science.1196380 21164016 PMC7211344

[bib8] Clegg N. J. Honda B. M. Whitehead I. P. Grigliatti T. A. Wakimoto B., 1998 Suppressors of position-effect variegation in *Drosophila melanogaster* affect expression of the heterochromatic gene *light* in the absence of a chromosome rearrangement. Genome 41: 495–503. 10.1139/g98-041 9796098

[bib9] Cook R. K. Christensen S. J. Deal J. A. Coburn R. A. Deal M. E., 2012 The generation of chromosomal deletions to provide extensive coverage and subdivision of the *Drosophila melanogaster* genome. Genome Biol. 13: R21 10.1186/gb-2012-13-3-r21 22445104 PMC3439972

[bib10] Coulthard A. B. Alm C. Cealiac I. Sinclair D. A. Honda B. M., 2010 Essential loci in centromeric heterochromatin of *Drosophila melanogaster*. I: The right arm of chromosome 2. Genetics 185: 479–495. 10.1534/genetics.110.117259 20382826 PMC2881131

[bib11] Coulthard A. B. Eberl D. F. Sharp C. B. Hilliker A. J., 2003 Genetic analysis of the second chromosome centromeric heterochromatin of *Drosophila melanogaster*. Genome 46: 343–352. 10.1139/g03-010 12834049

[bib12] Dietzl G. Chen D. Schnorrer F. Su K. C. Barinova Y., 2007 A genome-wide transgenic RNAi library for conditional gene inactivation in *Drosophila*. Nature 448: 151–156. 10.1038/nature05954 17625558

[bib14] Dimitri P. Caizzi R. Giordano E. Accardo M. C. Lattanzi G., 2009 Constitutive heterochromatin: a surprising variety of expressed sequences. Chromosoma 118: 419–435. 10.1007/s00412-009-0211-y 19412619

[bib15] Eberl D. F. Duyf B. J. Hilliker A. J., 1993 The role of heterochromatin in the expression of a heterochromatic gene, the rolled locus of *Drosophila melanogaster*. Genetics 134: 277–292. 8514136 10.1093/genetics/134.1.277 PMC1205430

[bib16] Eissenberg J. C. James T. C. Foster-Hartnett D. M. Hartnett T. Ngan V., 1990 Mutation in a heterochromatin-specific chromosomal protein is associated with suppression of position-effect variegation in *Drosophila melanogaster*. Proc. Natl. Acad. Sci. USA 87: 9923–9927. 10.1073/pnas.87.24.9923 2124708 PMC55286

[bib17] Eissenberg J. C. Reuter G., 2009 Cellular mechanism for targeting heterochromatin formation in *Drosophila*, pp. 1–47 in International Review of Cell and Molecular Biology, Vol. 273 chap 1., edited by Jeon K. W. Academic Press, Cambridge, MA. 10.1016/S1937-6448(08)01801-7 19215901

[bib18] Fitzpatrick K. A. Sinclair D. A. Schulze S. R. Syrzycka M. Honda B. M., 2005 A genetic and molecular profile of third chromosome centric heterochromatin in *Drosophila melanogaster*. Genome 48: 571–584. 10.1139/g05-025 16094423

[bib19] Hallson G. Syrzycka M. Beck S. A. Kennison J. A. Dorsett D., 2008 The *Drosophila* cohesin subunit Rad21 is a trithorax group (trxG) protein. Proc. Natl. Acad. Sci. USA 105: 12405–12410. 10.1073/pnas.0801698105 18713858 PMC2527924

[bib20] Hallson G. Hollebakken R. E. Li T. Syrzycka M. Kim I., 2012 dSet1 is the main H3K4 Di- and tri-methyltransferase throughout *Drosophila* development. Genetics 190: 91–100. 10.1534/genetics.111.135863 22048023 PMC3249358

[bib21] Heitz E., 1928 Das heterochromatin der moose, Bornträger, Berlin.

[bib22] Heitz E., 1929 *Heterochromatin*, *chromocentren*, *chromomeren. Komm*, Fischer, Jena, Germany.

[bib23] Hilliker A. J. Appels R., 1982 Pleiotropic effects associated with the deletion of heterochromatin surrounding rDNA on the X chromosome of *Drosophila*. Chromosoma 86: 469–490. 10.1007/BF00330122 6816533

[bib24] Hilliker A. J. Chovnick A. Clark S. H., 1981 The relative mutabilities of vital genes in *Drosophila melanogaster. Drosophila*. Inform. Serv. 56: 64–65.

[bib25] Hilliker A. J. Holm D. G., 1975 Genetic analysis of the proximal region of chromosome 2 of *Drosophila melanogaster*. I. Detachment products of compound autosomes. Genetics 81: 705–721. 814038 10.1093/genetics/81.4.705 PMC1213429

[bib26] Hilliker A. J., 1976 Genetic analysis of the centromeric heterochromatin of chromosome 2 of *Drosophila melanogaster*: deficiency mapping of EMS-induced lethal complementation groups. Genetics 83: 765–782. 823073 10.1093/genetics/83.4.765 PMC1213550

[bib27] Hoskins, R. A., C. D. Smith, J. W. Carlson, A. B. Carvalho, A. Halpern *et al.*, 2002 Heterochromatic sequences in a *Drosophila* whole-genome shotgun assembly. Genome Biology 3: RESEARCH0085.1–0085.6. 10.1186/gb-2002-3-12-research0085 PMC151187 12537574

[bib28] Hoskins R. A. Carlson J. W. Kennedy C. Acevedo D. Evans-Holm M., 2007 Sequence finishing and mapping of *Drosophila melanogaster* heterochromatin. Science 316: 1625–1628. 10.1126/science.1139816 17569867 PMC2825053

[bib29] Hoskins R. A. Carlson J. W. Wan K. H. Park S. Mendez I., 2015 The Release 6 reference sequence of the *Drosophila melanogaster* genome. Genome Res. 25: 445–458. 10.1101/gr.185579.114 25589440 PMC4352887

[bib30] Howe M. Dimitri P. Berloco M. Wakimoto B. T., 1995 Cis-effects of heterochromatin on heterochromatic and euchromatic gene activity in *Drosophila melanogaster*. Genetics 140: 1033–1045. 7672575 10.1093/genetics/140.3.1033 PMC1206659

[bib31] Kania A. Bellen H., 1995 Mutations in neuromusculin, a gene encoding a cell Roux Arch. Dev. Biol. 204: 259–270. 10.1007/BF00208493 28306121

[bib32] Kania A. Han P. L. Kim Y. T. Bellen H., 1993 Neuromusculin, a *Drosophila* gene expressed in peripheral neuronal precursors and muscles, encodes a cell adhesion molecule. Neuron 11: 673–687. 10.1016/0896-6273(93)90078-6 8398154

[bib33] Kharchenko P. V. Alekseyenko A. A. Schwartz Y. B. Minoda A. Riddle N. C., 2011 Comprehensive analysis of the chromatin landscape in *Drosophila melanogaster*. Nature 471: 480–485. 10.1038/nature09725 21179089 PMC3109908

[bib34] Koryakov D. E. Zhimulev I. F. Dimitri P., 2002 Cytogenetic analysis of the third chromosome heterochromatin of *Drosophila melanogaster*. Genetics 160: 509–517. 11861557 10.1093/genetics/160.2.509 PMC1461961

[bib35] Koryakov D. E. Domanitskaya E. V. Belyakin S. N. Zhimulev I. F., 2003 Abnormal tissue-dependent polytenization of a block of chromosome 3 pericentric heterochromatin in *Drosophila melanogaster*. J. Cell Sci. 116: 1035–1044. 10.1242/jcs.00283 12584247

[bib36] Koundakjian E. J. Cowan D. M. Hardy R. W. Becker A. H., 2004 The Zuker Collection: a resource for the analysis of autosomal gene function in *Drosophila melanogaster*. Genetics 167: 203–206. 10.1534/genetics.167.1.203 15166147 PMC1470872

[bib37] Larkin M. A. Blackshields G. Brown N. P. Chenna R. Mcgettigan P. A., 2007 Clustal W and Clustal X version 2.0. Bioinformatics 23: 2947–2948. 10.1093/bioinformatics/btm404 17846036

[bib38] Li C. Vagin V. V. Lee S. Xu J. Ma S., 2009 Collapse of germline piRNAs in the absence of argonaute3 reveals somatic piRNAs in flies. Cell 137: 509–521. 10.1016/j.cell.2009.04.027 19395009 PMC2768572

[bib39] Lin J. M. Kilman V. L. Keegan K. Paddock B. Emery-Le M., 2002 A role for casein kinase 2alpha in the *Drosophila* circadian clock. Nature 420: 816–820. 10.1038/nature01235 12447397

[bib40] Lindsley D. L. Hardy R. W., 1992 Cytology of *In(3L)TM8* and *In(3L)TM9*. DIS 71: 154.

[bib41] Lu B. Y. Emtage P. C. Duyf G. J. Hilliker A. J. Eissenberg J. C., 2000 Heterochromatin protein 1 is required for the normal expression of two heterochromatin genes in *Drosophila*. Genetics 155: 699–708. 10835392 10.1093/genetics/155.2.699 PMC1461102

[bib42] Lohe A. R. Hilliker A. J. Roberts P. A., 1993 Mapping simple repeated DNA sequences in heterochromatin of Drosophila melanogaster. Genetics 134: 1149–1174. 8375654 10.1093/genetics/134.4.1149 PMC1205583

[bib43] Marchant G. E. Holm D. G., 1988 a Genetic analysis of the heterochromatin of chromosome 3 in *Drosophila melanogaster*. I. Products of compound autosome detachment. Genetics 120: 503–517. 17246480 10.1093/genetics/120.2.503 PMC1203528

[bib44] Marchant G. E. Holm D. G., 1988 b Genetic analysis of the heterochromatin of chromosome 3 in *Drosophila melanogaster*. II. Vital loci identified through EMS mutagenesis. Genetics 120: 519–532. 17246481 10.1093/genetics/120.2.519 PMC1203529

[bib45] Marygold S. J. Roote J. Reuter G. Lambertsson A. Ashburner M., 2007 The ribosomal protein genes and minute loci of *Drosophila melanogaster*. Genome Biol. 8: R216 10.1186/gb-2007-8-10-r216 17927810 PMC2246290

[bib46] Mével-Ninio M. Pelisson A. Kinder J. Campos A. R. Bucheton A., 2007 The flamenco locus controls the gypsy and ZAM retroviruses and is required for *Drosophila* oogenesis. Genetics 175: 1615–1624. 10.1534/genetics.106.068106 17277359 PMC1855114

[bib47] Miklos G. L. G. Rubin G. M., 1996 The role of the genome project in determining gene function: insights from model organisms. Cell 86: 521–529. 10.1016/S0092-8674(00)80126-9 8752207

[bib48] Mukai T., 1964 The genetic structure of natural populations of *Drosophila melanogaster*. I. Spontaneous mutation rate of polygenes controlling viability. Genetics 50: 1–19. 14191352 10.1093/genetics/50.1.1 PMC1210633

[bib49] Myster S. H. Wang F. Cavallo R. Christian W. Bhotika S., 2004 Genetic and bioinformatic analysis of 41C and the 2R heterochromatin of *Drosophila melanogaster*: a window on the heterochromatin-euchromatin junction. Genetics 166: 807–822. 10.1534/genetics.166.2.807 15020470 PMC1470754

[bib50] Ohnishi T. Ohnishi S. T. Salerno J. C. 2018 Five decades of research on mitochondrial NADH-quinone oxidoreductase (complex I). J. Biol Chem. Oct 25: 1249–1264. 10.1515/hsz-2018-0164 30243012

[bib51] Reuter G. Giarre M. Farah J. Gausz J. Spierer A., 1990 Dependence of position-effect variegation in *Drosophila* on dose of a gene encoding an unusual zinc-finger protein. Nature 344: 219–223. 10.1038/344219a0 2107402

[bib52] Riddle N. C. Elgin S. C. R., 2018 The *Drosophila* Dot Chromosome: Where Genes Flourish Amidst Repeats. Genetics 210: 757–772. 10.1534/genetics.118.301146 30401762 PMC6218221

[bib53] Riddle N. C. Minoda A. Kharchenko P. V. Alekseyenko A. A. Schwartz Y. B., 2011 Plasticity in patterns of histone modifications and chromosomal proteins in *Drosophila* heterochromatin. Genome Res. 21: 147–163. 10.1101/gr.110098.110 21177972 PMC3032919

[bib54] Rossi A. Kontarakis Z. Gerri C. Nolte H. Hölper S., 2015 Genetic compensation induced by deleterious mutations but not gene knockdowns. Nature 524: 230–233. 10.1038/nature14580 26168398

[bib55] Rossi F. Moschetti R. Caizzi R. Corradini N. Dimitri P., 2007 Cytogenetic and molecular characterization of heterochromatin gene models in *Drosophila melanogaster*. Genetics 175: 595–607. 10.1534/genetics.106.065441 17110485 PMC1800633

[bib56] Sarpal R. Pellikka M. Patel R. R. Hui F. Y. Godt D., 2012 Mutational analysis supports a core role for *Drosophila* α-catenin in adherens junction function. J. Cell Sci. 125: 233–245. 10.1242/jcs.096644 22266901

[bib57] Schotta G. Lachner M. Sarma K. Ebert A. Sengupta R., 2004 A silencing pathway to induce H3–K9 and H4–K20 trimethylation at constitutive heterochromatin. Genes Dev. 18: 1251–1262. 10.1101/gad.300704 15145825 PMC420351

[bib58] Schulze S. Sinclair D. A. Silva E. Fitzpatrick K. A. Singh M., 2001 Essential genes in proximal 3L heterochromatin of *Drosophila melanogaster*. Mol. Gen. Genet. 264: 782–789. 10.1007/s004380000367 11254125

[bib59] Schulze S. R. Sinclair D. A. Fitzpatrick K. A. Honda B. M., 2005 A genetic and molecular characterization of two proximal heterochromatic genes on chromosome 3 of *Drosophila melanogaster*. Genetics 169: 2165–2177. 10.1534/genetics.103.023341 15687284 PMC1449577

[bib60] Shi M. Yue Z. Kuryatov A. Lindstrom J. M. Sehgal A., 2014 Identification of Redeye, a new sleep-regulating protein whose expression is modulated by sleep amount. eLife 3: e01473 10.7554/eLife.01473 24497543 PMC3912633

[bib61] Sinclair D. A. R. Schulze S. Silva E. Fitzpatrick K. A. Honda B. M., 2000 Essential Genes in Autosomal Heterochromatin of *Drosophila melanogaster*. Genetica 109: 9–18. 10.1023/A:1026500620158 11293800

[bib62] Sinclair D. A. R. Syrzycka M. Macauley M. S. Rastgardani T. Komljenovic I., 2009 *Drosophila* O-GlcNAc transferase (OGT) is encoded by the *Polycomb* group (PcG) gene, *super sex combs* (*sxc*). Proc. Natl. Acad. Sci. USA 106: 13427–13432. 10.1073/pnas.0904638106 19666537 PMC2726349

[bib63] Smith C. D. Shu S. Q. Mungall C. J. Karpen G. H., 2007 The release 5.1 annotation of *Drosophila melanogaster* heterochromatin. Science 316: 1586–1591. 10.1126/science.1139815 17569856 PMC2819280

[bib64] Tschiersch B. Hofmann A. Krauss V. Dorn R. Korge G., 1994 The protein encoded by the *Drosophila* position-effect variegation suppressor gene *Su(var)3–9* combines domains of antagonistic regulators of homeotic gene complexes. EMBO J. 13: 3822–3831. 10.1002/j.1460-2075.1994.tb06693.x 7915232 PMC395295

[bib65] Vass S. Cotterill S. Valdeolmillos A. M. Barbero J. L. Lin E., 2003 Depletion of Drad21/Scc1 in *Drosophila* cells leads to instability of the cohesin complex and disruption of mitotic progression. Curr. Biol. 13: 208–218. 10.1016/S0960-9822(03)00047-2 12573216

[bib66] Vilinsky I. Stewart B. A. Drummond J. Robinson I. Deitcher D. L., 2002 A *Drosophila SNAP-25* null mutant reveals context-dependent redundancy with *SNAP-24* in neurotransmission. Genetics 162: 259–271. 12242238 10.1093/genetics/162.1.259 PMC1462260

[bib67] Wakimoto B. T. Hearn M. G., 1990 The effects of chromosome rearrangements on the expression of heterochromatic genes in chromosome 2L of Drosophila melanogaster. Genetics 125: 141–154. 2111264 10.1093/genetics/125.1.141 PMC1203996

[bib70] Yan D. Neumüller R. A. Buckner M. Ayers K. Li H. Hu Y. Yang-Zhou D., 2014 A regulatory network of Drosophila germline stem cell self-renewal. Developmental Cell 28: 459–473. 10.1016/j.devcel.2014.01.020 PMC3998650 24576427

[bib68] Yoshiyama T. Namiki T. Mita K. Kataoka H. Niwa R. 2006 Neverland is an evolutionally conserved Rieske-domain protein that is essential for ecdysone synthesis and insect growth. Development 133: 2565–74. 10.1242/dev.02428 16763204

[bib69] Zhang P. Spradling A. C., 1994 Insertional mutagenesis of *Drosophila* heterochromatin with single *P* elements. Proc. Natl. Acad. Sci. USA 91: 3539–3543. 10.1073/pnas.91.9.3539 8170943 PMC43615

